# Comparison Between Radiographic Vertebral Left Atrial Size (VLAS) and Echocardiographic Methods for Predicting Left Atrial Remodeling in Dogs With Mitral Valve Disease

**DOI:** 10.1155/vmi/5516116

**Published:** 2025-02-06

**Authors:** Natália Babolim Pereira, Guilherme Andraus Bispo, Maurício Peres Carneiro, Ricardo de Souza Buzo, Daniela Ribas Jané, Laís Calazans Menescal Linhares, Paulo Sergio Patto dos Santos, Luciana Del Rio Pinoti, Wagner Luis Ferreira

**Affiliations:** ^1^Department of Clinical, Surgery and Animal Reproduction, School of Veterinary Medicine, São Paulo State University (UNESP), Araçatuba, São Paulo, Brazil; ^2^Department of Veterinary Clinic and Surgery, School of Agricultural and Veterinary Sciences, São Paulo State University (UNESP), Jaboticabal, São Paulo, Brazil

**Keywords:** atrial remodeling, degeneration, endocardiosis, MINE score, VLAS

## Abstract

**Objective:** To investigate whether there is a positive correlation between vertebral left atrial size (VLAS) and echocardiographic variables—left atrium-to-aorta ratio (LA/Ao), left ventricular fractional shortening (FS), left ventricular internal diameter in diastole normalized to body weight (LVIDdn), and left ventricular early filling velocity (E-wave)—and the Mitral INsufficiency Echocardiographic (MINE) echocardiographic score.

**Study Design:** Prospective randomized blinded study.

**Animals:** A total of 26 dogs.

**Methods:** Dogs diagnosed with MVD participated in the study. All patients underwent radiographic and echocardiographic evaluation.

**Results:** A high correlation strength could be observed between the left atrial enlargement predictor VLAS and the LA/Ao ratio (88%), as well as with LVIDdn (75%) and E-wave (74%). Furthermore, a correction strength of 84% was verified between VLAS and the MINE score. The analysis of the FS variable did not demonstrate a statistically significant relationship (*p* value of 0.06).

**Conclusions and Clinics Relevance:** VLAS has a positive relationship and important correlation with the echocardiographic variables and MINE score. Associated with its higher accessibility in clinical routine is a potential diagnostic method to detect left atrial enlargement in dogs, contributing to the diagnostic conduct in DVM.

## 1. Introduction

Mitral valve disease (MVD) is the main cause of congestive heart failure in dogs [1–3]. It is histopathologically characterized by the progressive fragmentation and modification of the arrangement of collagen and elastin in the extracellular matrix of heart valve tissue and the accumulation of mucopolysaccharides, causing its degeneration and insufficiency [2, 4, 5]. Therefore, it leads to left atrial enlargement because of the chronicity and severity of blood regurgitation [6].

Measuring this heart chamber allows the prediction of the risk of developing congestive heart failure and the estimation of the prognosis of the condition [1, 7–10]. In this aspect, Vezzosi et al. [11] proposed an MVD severity classification. This classification, called the Mitral INsufficiency Echocardiographic (MINE) score, has been objective and effective in providing prognostic information to patients through echocardiographic examination.

This examination is the gold standard non-invasive diagnostic method for evaluating atrial size, as well as for diagnosing MVD [1]. However, the feasibility of performing echocardiography may be compromised due to the critical clinical condition of patients, precarious equipment, and the low level of knowledge required to perform the examination and interpret the results, in addition to being financially inaccessible for some guardians [12, 13]. In contrast, radiographic examination is a widely accessible and more economically viable method [1, 14]. The best-known methods to estimate heart size using radiography are vertebral heart size (VHS) and vertebral left atrial size (VLAS). Currently, VHS, proposed by Buchanan and Bücheler [15], is the most used technique and allows a general assessment of heart size but does not provide specific information, such as whether the right or left antimere is more affected or which heart chamber is more increased, information that is essential for prescribing appropriate treatment [1].

In contrast, VLAS is a recently proposed tool for radiographic measurement of the left atrium in dogs. It is considered a useful and repeatable predictor for evaluating the remodeling of this heart chamber [16, 17]. The literature shows a growing number of studies supporting the diagnostic utility of VLAS to detect left atrial enlargement, corroborated by echocardiography, and clinical staging in dogs with mitral valve disease [12, 13, 18, 19]. However, unlike VHS, knowledge about the clinical usefulness of VLAS for detecting left atrial remodeling is limited, making further studies necessary to evaluate its diagnostic accuracy in patients with MVD compared with echocardiography.

Considering this lack of data, this study aimed to investigate the correlation between VLAS and echocardiographic variables, predictors of left atrial remodeling and congestion: left atrium-to-aorta ratio (LA/Ao), left ventricular fractional shortening (FS), left ventricular internal diameter in diastole normalized to body weight (LVIDdn), and left ventricular early filling velocity (E wave) beyond VLAS correction with the MINE echocardiographic score. It is assumed that there will be a positive correlation between the VLAS and the echocardiographic variables mentioned above.

## 2. Materials and Methods

### 2.1. Animals

This is a prospective randomized blinded study that was approved by the local ethics committee (process no. 0856-2021). Twenty-six male and female dogs diagnosed with MVD participated in the study. All patients included in the study were treated in the cardiology sector of the Luiz Quintiliano de Oliveira Veterinary Hospital of the São Paulo State University, School of Veterinary Medicine of Araçatuba (FMV–UNESP) from December 2021 to October 2022.

The inclusion criteria were the presence of systolic murmur of any grade, identification and complete anamnesis with information regarding clinical symptoms, and the use or not of drugs to treat MVD.

Exclusion criteria: nonvisualization of the caudal vena cava in the radiography examination, presence of vertebral abnormalities, and echocardiographic and radiographic examinations within more than 24 h between them. The caudal vena cava was not visualized on two radiographic examinations. Thus, two dogs were excluded.

### 2.2. Data Collection

Clinical records were used to collect data on breed, weight, size, sex, age, presence of clinical symptoms (cough, easy tiredness, cyanosis, dyspnea, and/or syncope), and use or not of drugs to treat MVD (enalapril/benazepril, pimobendan, spironolactone, and/or furosemide). The dogs were classified in terms of size as described by Cortopassi and Conti-Patara [20] as small size (weighing up to 10 kg), medium size (between 10.1 and 25 kg), and large size (more than 25.1 kg).

The presence of a heart murmur in the mitral focus was verified during the physical examination. It was classified in all animals by the same professional from the cardiology sector, thus avoiding a possible bias in the study regarding its classification.

### 2.3. Echocardiographic Study

The echocardiographic examination was double-blindly performed, in which the operator was unaware of the dogs' radiographic findings. Furthermore, it was prepared by the same operator, avoiding bias due to a change in the evaluator.

Shaving was performed between the third and fifth right and left intercostal spaces of the dogs. Subsequently, they were positioned on an appropriate foam mattress in the right lateral recumbency position to acquire images in the right parasternal view, followed by the left lateral recumbency position to obtain images in the left parasternal view. The echocardiographic images were obtained using the Essaote Mylab 30 Gold echocardiographic equipment, with multifrequency transducers of 3–8 MHz (dogs under 10 kg) and 1–4 MHz (dogs over 10 kg), with simultaneous electrocardiographic recording and using gel to create an interface between the transducer and the dogs' chest, by the same evaluator (GAB).

**Figure 1:**
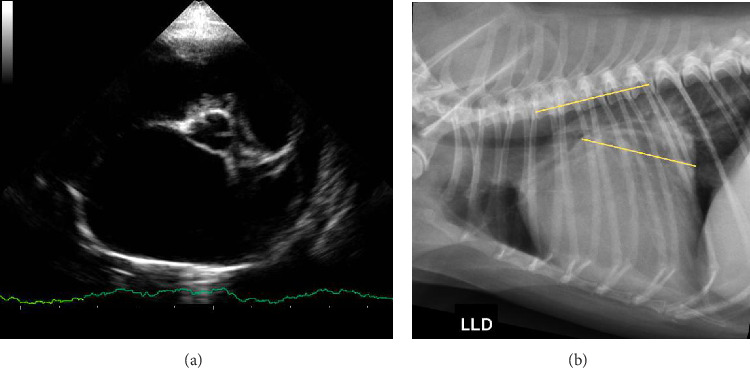
Echocardiographic and radiographic images of a canine patient classified as stage C and severe MVD, according to the ACVIM classification and MINE score, respectively. (a) Echocardiographic image taken in the right parasternal view of the transverse axis at the level of the left atrium to obtain the LA/Ao ratio; evident left atrial remodeling is noted with an increase in the LA/Ao ratio. (b) Plain radiographic image in the right laterolateral projection of the patient's thoracic region showing the location of the VLAS measurement.

Echocardiographic images of the short axes of the left ventricle were obtained in the right parasternal view in the chordal plane, and the final systolic and diastolic diameters and volumes were obtained by the Teichholz method using the M mode. It allowed calculating the left ventricular fractional shortening (FS) and the left ventricle internal diameter in diastole (LVIDd), which was LVIDdn, according to the following equation: LVIDd/WEIGHT(kg)^0.294^ [8]. The LA/Ao was also obtained in the transverse section of the left ventricle but at the height of the left atrium [21], as shown in Figure 1(a).

Then, the dogs were positioned in left lateral recumbency, and in four chambers' view were obtained the E wave, using the pulsed Doppler [22]. The images were stored and analyzed by the same operator after carrying out the echocardiographic study.

All dogs were classified according to the MVD staging proposed by ACVIM into stages A, B (B1 and B2), C, and D [1].

Furthermore, it was classified according to the echocardiographic score of MVD severity, called the MINE score, proposed by Vezzosi et al. [11]. To this end, scores related to the cutoff values of the echocardiographic variables LA/Ao, LVIDdn, FS, and E-wave were used. Subsequently, the score and the acquired value were added, suggesting the categorization of MVD in mild (4–5 points), moderate (6–7 points), severe (8–12 points), and final stages (13–14 points).

### 2.4. Radiography Study

As in the echocardiographic study, the radiographic examination was performed by the same operator, who was unaware of the echocardiographic findings and clinical characteristics of the patients.

A simple radiography of the thoracic region of the dogs was performed in the right lateral projection. Then, VLAS was measured according to the technique proposed by Malcolm et al. [18]. A line was drawn from the center of the most ventral part of the carina to the most caudal part of the left atrium, where it intersects with the dorsal portion of the caudal vena cava to measure the left atrium based on this technique. This line was positioned parallel to the thoracic vertebrae, starting at the fourth vertebra of this segment. Thus, the measurement trace was obtained, as shown in Figure 1(b). VLAS was estimated in number of vertebral bodies from the second line drawn, based on the vertebral body length closest to 0.1.

The radiographic images were taken using an SHF 730 fixed radiographic unit (CRX, Brazil), with a capacity of 500 mA and 150 kVp, with an exposure time of 0.05–5 s. The right laterolateral view was used for thoracic assessment, complying with radiological protection standards. The images and heart measurements of the radiographic examinations were performed on the screen of the computerized radiology system—CR (Carestream).

### 2.5. Statistical Analysis

Statistical analysis was performed using the free statistical software R v4.1.0 [23]. To this end, a linear regression that had as predictors the echocardiographic variables LA/Ao, LVIDdn, FS, and E-wave, and MINE score which were compared with the VLAS variable obtained radiographically to identify the existence of relationship between them, was performed.

A percentage analysis was conducted to outline the profile of the animals. Descriptive statistics were also performed, obtaining the mean, standard deviation, standard error of the mean, and upper and lower limits of the echocardiographic and radiographic variables, with the values obtained in each stage of the MINE score classification. Finally, a correlation plot was created to compare the obtained variables (radiographic and echocardiographic) throughout the study to observe more clearly the existence of a relationship (positive or negative) between them.

The results were considered significant when the *p* value < 0.05.

## 3. Results

Twenty-six dogs participated in the study, 54% females and 46% males, with a higher predominance of small dogs (88.5%), followed by medium-sized dogs (11.5%) and the absence of large dogs. There was a predominance of mixed breed animals (*n* = 11), and the others were Shih Tzu (*n* = 5), Poodle (*n* = 4), Lhasa Apso (*n* = 2), Maltese (*n* = 2), Dachshund (*n* = 1), and Schnauzer (*n* = 1).

Regarding age, the animals were between 5 and 15 years old, with a predominance of those aged 14 years, which contained a higher number of individuals (six dogs) compared with the others. Moreover, dogs over 11 years of age were the majority, constituting 80.7% of the sample.

The grade of heart murmur in the mitral focus was obtained from the most to the least prevalent: grade III present in eight animals (31%), grade IV in seven animals (27%), grade V in six (23%), grade VI in four dogs (15%), and grade II in one individual (4%).

The symptomatic population was 14 dogs (54%) and the asymptomatic dogs consisted of 12 animals (46%). The reported clinical signs, concomitant or not, were dyspnea, cough, tiredness with minimal effort, and syncope. Moreover, eight (31%) out of the 26 dogs that constituted the sample were continuously using drugs recommended to treat MVD. The animals received enalapril/benazepril, pimobendan, furosemide, and/or spironolactone. Importantly, five out of the eight animals were simultaneously using these four drugs. Also, two animals received pimobendan and enalapril/benazepril, and one used only enalapril/benazepril.

According to the DVM classification by ACVIM, half of the sample group (50%) fell into stage C of the disease. A total of 35% was classified as stage B1, 11% as B2, and 4% as stage D. Furthermore, they were classified according to the echocardiographic severity score of MVD (MINE score), showing a higher percentage of individuals with severe MVD (46%), followed by those with mild (42%), moderate (8%), and end-stage MVD (4%).

The distribution of values obtained by echocardiographic and radiographic analyses was analyzed to perform descriptive statistics, as detailed in Table 1. Furthermore, Table 2 describes the evaluation based on the distribution of data according to the stage of classification of the MINE score.

Linear regression showed a positive relationship between VLAS and LA/Ao by the *p* value < 0.001, with a strength of 77%. The test also indicated this relationship between VLAS and E-wave (*p* value < 0.001), with a strength of 55%. In addition, the same was observed between VLAS and LVIDdn, with a *p* value < 0.001 and a strength of 56%. The analysis of the FS variable did not demonstrate a statistically significant relationship (*p* value of 0.06).

**Figure 2:**
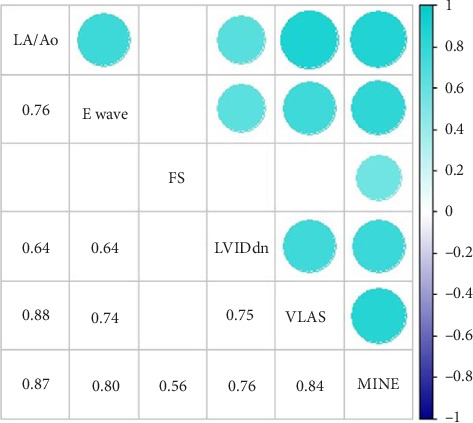
Correlation matrix between echocardiographic and radiographic variables and MINE score of dogs (*n* = 26) with MVD. Abbreviations: E wave, left ventricular early filling velocity; FS, left ventricular fractional shortening; LA/Ao, left atrium-to-aorta ratio; LVIDdn, left ventricular internal diameter in diastole normalized to body weight; MINE, MINE score; VLAS, vertebral left atrial size.

The results enabled the construction of a correlation plot between the analyzed variables (Figure 2). A high correlation strength could be observed between the left atrial enlargement predictor VLAS and the LA/Ao ratio (88%), as well as with LVIDdn (75%) and E-wave (74%). Furthermore, a correction strength of 84% was verified between VLAS and the MINE score. Also, echocardiographic measurements showed a positive correlation with each other. The value of the estimated regression coefficient and the standard error are described in Table 3.

## 4. Discussion

This study shows a strong correlation between echocardiographic variables and VLAS, indicating the radiographic predictor as a potential diagnostic method to identify left atrial remodeling in dogs.

The present study had its sample mainly composed of small dogs (up to 10 kg of body weight), with a mean age of 11.9 years. The data are corroborated by the literature, which describes animals of this size as the main representatives of MVD cases and emphasizes the age predisposition in older individuals [1, 24–26]. The incidence of females in the current research was slightly higher than that of males. Studies have shown that males are more affected, which differs from our results, although the number of affected females has been increasing in the literature [1, 24, 27–29]. The very similar number of females to that of males, associated with the small sample size, shows that the existence of a predilection regarding the sex of the animal could not be confirmed by this research.

A heart murmur in the mitral focus was identified in the entire sample group, classified from grade II/VI to VI/VI. Furthermore, the simultaneous analysis of the classification of the MINE score and that proposed by ACVIM shows that all animals in stage B1 were classified with a mild MVD by the MINE score. All dogs in stage B2 were classified as severe by MINE. Two (15.3%) of those patients classified as stage C by ACVIM were in the mild stage of the MINE score, two were in the moderate stage, and the remaining nine (69.2%) had severe MVD. Finally, the only individual classified in stage D had MVD in the final stage.

The VLAS radiographic predictor demonstrated a positive relationship and important correlation strength with the analyzed echocardiographic parameters, which is in line with previous studies [12, 13, 18, 30, 31]. Among them, a positive relationship and strong correlation strength with LA/Ao could be observed. It indicates that the left atrial enlargement identified by the echocardiographic examination can also be identified by the radiographic method. Therefore, the higher the LA/Ao, the higher the corresponding VLAS. The literature corroborates this information, as other authors have obtained similar results [11, 12, 18, 30, 31].

The LVIDdn was also positively related to VLAS, which corresponds to the findings of recent studies on the diagnostic accuracy of this radiographic method [12, 30, 31]. According to Cornell et al. [32], LVIDdn evaluates left ventricular function and remodeling, being important in the evaluation of MVD, as it is associated not only with left atrial dilation but also ventricular enlargement. Theoretically, VLAS is a predictor of left atrial remodeling, but findings documented by Micawa et al. [30] indicated that its increase may be indirectly associated with left ventricular remodeling. As reported by these authors, the results suggest that VLAS reflects the enlargement of the entire heart and not just the left atrium. In fact, the measurement corresponds to the distance between the carina and the caudal edge of the left atrium where it crosses the caudal vena cava. Thus, the trachea is displaced dorsally with heart remodeling, increasing the distance between the vena cava and the carina, resulting in a corresponding increase in VLAS [30].

The two echocardiographic variables (LA/Ao and LVIDdn) were those of choice in studies that aimed to evaluate the diagnostic accuracy of VLAS, especially due to its high specificity and sensitivity in identifying atrial remodeling in dogs. These requirements were not subject to evaluation in the present study but the literature has included positive results, namely, Stepien et al. [19] identified a sensitivity of 40% and a specificity of 96% in the cutoff value for VLAS ≥ 3 vertebrae, Vezzosi et al. [13] reported sensitivity of 66% and specificity of 100% and limited false positive results with the latter, Duler et al. [12] identified a rate of 26% of false positives and 10% of false negatives, and Levicar et al. [31] found sensitivity of 72% and specificity of 78%. The data also reinforce the results obtained in the present study regarding the strength of correlation and positive relationship between VLAS and the variables LA/Ao and LVIDdN, supporting the effectiveness of this radiographic method in identifying left atrial enlargement.

Unlike previous research, this study statistically analyzed other echocardiographic variables searching for possible correlations with the evaluated radiographic method. The correlation found between VLAS and E wave stands out. According to Petric [33], the increase in this parameter is directly related to atrial remodeling, as an increase in left heart chambers promotes an increase in the filling pressure of the left ventricle and, consequently, the E wave.

This is the first study to our knowledge that assesses the existence of a relationship between VLAS and MINE score, which becomes understandable because the classification of MVD by the MINE score is related to the echocardiographic variables. In this context, the higher the values of the variables predicting atrial and left ventricular remodeling, the greater the MVD severity attributed to the MINE score, and a corresponding increase in VLAS can be identified.

On the other hand, FS, not evaluated in previous research, demonstrated no statistically significant relationship with VLAS, and a moderate correlation strength was identified (56%). Physiologically, mitral regurgitation with heart remodeling causes the left ventricle to enter a hyperdynamic state with volume overload and increased sympathetic tone [33, 34]. This left ventricular response is directly related to FS, which remains at normal values or even increases until there is left ventricular systolic dysfunction. At this point, VLAS did not demonstrate a relationship with FS because there were no dogs with systolic dysfunction in the present study, keeping this value within normal limits.

However, Duler et al. [12] highlighted that VLAS has inherent limitations that must be considered when choosing this diagnostic method. An example is the difficulty in identifying the cardiac silhouette in some radiographic projections, preventing the measurement from being properly performed. Furthermore, small variations in VLAS measurement can have a large clinical impact due to its relatively limited scale. However, the data mentioned above and corroborated by the literature indicate VLAS as a very advantageous method for predicting left atrial remodeling, with a high correlation with the echocardiographic variables studied here, considered the gold standard in identifying remodeling of the heart chambers.

This study has some limitations, such as including only dogs with mitral valve disease, and the findings for other heart diseases should not be interchanged. In addition, it only includes the echocardiographic variables that make up the MINE score. Another limitation could be the sample size. Without a proper power analysis or sample size determination study, there is a risk that the sample might be underpowered. This limitation should be acknowledged in the study, and results should be interpreted with caution.

## 5. Conclusions

This study indicated that the radiographic predictor VLAS has a positive relationship and important correlation with the echocardiographic variables and, associated with its higher accessibility in clinical routine, is a potential diagnostic method to detect left atrial enlargement in dogs, contributing to the diagnostic conduct in DVM. Furthermore, it demonstrated a strong correlation with the MINE echocardiographic score.

## Figures and Tables

**Table 1:** Descriptive statistics (mean ± standard deviation, standard error of the mean, confidence interval, and upper and lower limits) of echocardiographic and radiographic variables of dogs with MVD (*n* = 26).

Variable	Mean ± standard deviation	Standard error of the mean	Confidence interval	Lower limit	Upper limit
LA/Ao	2.08 ± 0.85	0.77	1.77–2.4	1.15	4.25
LVIDdn	1.92 ± 0.73	0.14	1.62–2.22	1.2	4.68
FS (%)	48.61 ± 9.32	1.83	44.9–52.4	32	84
E wave (m/s)	1.09 ± 0.51	0.1	1.3–1.77	0.49	2.39
VLAS (vertebrae)	2.74 ± 0.85	0.17	2.4–3.08	1.7	4.8

*Note:* LVIDdN, left ventricular internal diameter in diastole normalized to body weight; SF, left ventricular shortening fraction; E wave, left ventricular early filling velocity.

Abbreviations: LA/Ao, left atrium-to-aorta ratio; VLAS, vertebral left atrial size.

**Table 2:** Descriptive statistics (mean ± standard deviation, standard error of the mean, confidence interval, and upper and lower limits) of echocardiographic and radiographic variables of dogs (*n* = 26) according to the MINE score classification.

Score of gravity	Variable	Mean ± SD	SE	CI	Lower limit	Upper limit
Mild (*n* = 11)	LA/Ao	1.41 ± 0.15	0.05	1.3–1.51	1.25	1.6
	LVIDdn	1.42 ± 0.12	0.04	1.34–1.5	1.2	1.68
	FS (%)	43.3 ± 5.58	1.68	40–47	32	52.3
	E-wave (m/s)	0.7 ± 0.14	0.04	0.61–0.8	0.49	0.94
	VLAS (vertebrae)	2.05 ± 0.25	0.07	1.88–2.2	1.7	2.6

Moderate⁣^∗^ (*n* = 2)	LA/Ao	1.86 ± 0.88	0.62	−6–9.7	1.24	2.48
	LVIDdn	1.49 ± 0.13	0.09	0.35–2.6	1.4	1.58
	FS (%)	46.5 ± 7.78	5.5	−23–116	41	52
	E-wave (m/s)	0.85 ± 0.41	0.3	−2.9–4.6	0.55	1.14
	VLAS (vertebrae)	2.35 ± 0.21	0.15	0.44–4.2	2.2	2.5

Severe (*n* = 12)	LA/Ao	2.66 ± 0.59	0.17	2.28–3	2.06	4.25
	LVIDdn	2.22 ± 0.36	0.11	1.99–2.4	1.33	2.6
	FS (%)	53.88 ± 10.2	2.94	47.4–60	44	84
	E-wave (m/s)	1.44 ± 0.48	0.14	1.1–1.74	0.69	2.39
	VLAS (vertebrae)	3.34 ± 0.72	0.2	2.88–3.8	2.4	4.8

Late stage⁣^∗∗^	—	—	—	—	—	—

*Note:* LVIDdn, left ventricular internal diameter in diastole normalized to body weight; FS, left ventricular shortening fraction; E-wave, left ventricular early filling velocity.

Abbreviations: CI, confidence interval; LA/Ao, left atrium-to-aorta ratio; SD, standard deviation; SE, Standard error of the mean; VLAS, vertebral left atrial size.

⁣^∗^Only two animals were classified with moderate-stage MVD, according to the MINE score, which justifies the discrepancy in the results.

⁣^∗∗^Only one animal was classified as having end-stage MVD, according to the MINE score. Therefore, descriptive statistics could not be performed.

**Table 3:** Estimated regression coefficients and standard error of echocardiographic variables of dogs (*n* = 26) in relation to the VLAS.

Variable	COEF	SE	*p* value
LA/Ao	0.96	0.11	< 0.001
LVIDdn	0.86	0.16	< 0.001
FS	0.03	0.02	0.06
E wave	1.23	0.23	< 0.001

*Note:* LVIDdn, left ventricular internal diameter in diastole normalized to body weight; E-wave, left ventricular early filling velocity; FS, left ventricular shortening fraction; COEF, regression coefficients.

Abbreviations: LA/Ao, left atrium-to-aorta ratio; SE, standard error; VLAS, vertebral left atria.

## Data Availability

The raw data supporting the conclusions of this study are available from the corresponding author upon reasonable request.
